# Nonsense-Mediated mRNA Decay and Loss-of-Function of the Protein Underlie the X-Linked Epilepsy Associated with the W356× Mutation in Synapsin I

**DOI:** 10.1371/journal.pone.0067724

**Published:** 2013-06-20

**Authors:** Maila Giannandrea, Fabrizia C. Guarnieri, Niels H. Gehring, Elena Monzani, Fabio Benfenati, Andreas E. Kulozik, Flavia Valtorta

**Affiliations:** 1 Division of Neuroscience, San Raffaele Scientific Institute and Vita-Salute University, Milan, Italy; 2 Institute for Genetics, University of Cologne, Cologne, Germany; 3 Department of Neuroscience and Brain Technologies, The Italian Institute of Technology, Genoa, Italy; 4 Department of Experimental Medicine, University of Genoa, Genoa, Italy; 5 Department of Pediatric Oncology, Hematology and Immunology, University of Heidelberg Medical Center and Molecular Medicine Partnership Unit, EMBL and University of Heidelberg, Heidelberg, Germany; University of Iowa Carver College of Medicine, United States of America

## Abstract

Synapsins are a family of neuronal phosphoproteins associated with the cytosolic surface of synaptic vesicles. Experimental evidence suggests a role for synapsins in synaptic vesicle clustering and recycling at the presynaptic terminal, as well as in neuronal development and synaptogenesis. Synapsin knock-out (*Syn1^−/−^*) mice display an epileptic phenotype and mutations in the *SYN1* gene have been identified in individuals affected by epilepsy and/or autism spectrum disorder. We investigated the impact of the c.1067G>A nonsense transition, the first mutation described in a family affected by X-linked syndromic epilepsy, on the expression and functional properties of the synapsin I protein. We found that the presence of a premature termination codon in the human *SYN1* transcript renders it susceptible to nonsense-mediated mRNA decay (NMD). Given that the NMD efficiency is highly variable among individuals and cell types, we investigated also the effects of expression of the mutant protein and found that it is expressed at lower levels compared to wild-type synapsin I, forms perinuclear aggregates and is unable to reach presynaptic terminals in mature hippocampal neurons grown in culture. Taken together, these data indicate that in patients carrying the W356× mutation the function of synapsin I is markedly impaired, due to both the strongly decreased translation and the altered function of the NMD-escaped protein, and support the value of *Syn1^−/−^* mice as an experimental model mimicking the human pathology.

## Introduction

Epileptic syndromes, including idiopathic epilepsies, have a large genetic component. In the last decades, mutations in various subunits of a number of ion channels - including voltage-gated channels, as well as GABA_A_, AMPA and nicotinic receptors - have been found to play a major role in the pathogenesis of several epileptic syndromes [1]–[3]. However, the number of potential genes whose mutation may underlie epilepsy is much larger. Among these, of particular interest are those involved in neuronal development and migration, synaptogenesis, neurotransmitter release and synaptic plasticity. Accordingly, large families of proteins involved in synaptic vesicle (SV) trafficking and exo-endocytosis (over 140 gene products) could be involved in epilepsy. Although a large number of these genes have been inactivated in animal models to uncover the physiological role of the respective encoded proteins, only few mutants have been reported to exhibit an epileptic phenotype. A severe epileptic phenotype was found in genetically altered mice lacking members of the synapsin (Syn) and SV2 families of SV proteins [4]–[7], whereas epilepsy was not observed in mouse mutants deleted for other SV or presynaptic plasma membrane proteins.

Syns are a family of neuronal phosphoproteins associated with the cytosolic surface of SVs. In mammals the family comprises three members encoded by distinct genes (*SYN1*, *SYN2* and *SYN3*, respectively located on chromosomes X, 3 and 22) that in turn give rise to various splicing isoforms. In the central nervous system, the vast majority of neurons expresses at least one Syn isoform [8]. However, the functions of these proteins are not fully understood to date. Experimental evidence suggests a role for Syns in SV clustering and recycling in the presynaptic terminal, as well as in neuronal development and synaptogenesis [9], [10]. Syn isoforms share highly conserved domains at the NH_2_-terminus (particularly domains A and C), while they differ in the COOH-terminal portion [8], [11]. The most studied member of the family is Syn I. Syn function is primarily regulated by site-specific phosphorylation, which leads to conformational changes and consequent modification in the biochemical properties of the protein. In particular, Syn I is the substrate of several kinases such as protein kinase A, Ca^2+^/calmodulin-dependent kinases I/II and IV, mitogen-activated protein kinase, Src and cyclin-dependent kinase 5, and thus represents a central downstream effector of many signalling pathways in the presynaptic terminal [12]–[15]. Indeed, in mature neurons Syn I is thought to regulate trafficking between the reserve pool and the readily-releasable pool of SVs through phosphorylation-dependent interactions with the actin cytoskeleton and the SV membrane [11]. Moreover, Syn I plays a role in the final post-docking steps of exocytosis, including SV priming and fusion [16], [17].

Mice lacking the *Syn* genes are viable and fertile but, starting from the second month of age, they exhibit an epileptic phenotype characterized by frequent sensory-evoked generalized tonic–clonic attacks [4], [5], [18]–[20]. Seizures are not observed in heterozygous female mice, pointing to a recessive pattern of inheritance. These observations suggest an involvement of Syns in the establishment of the delicate balance between inhibitory and excitatory transmission that controls cortical excitability. Interestingly, it was recently shown that the ablation of the *Syn* genes is associated with opposite changes in the strength of excitatory and inhibitory synapses and leads to hyperexcitability of cortical networks [18], [21]–[25], indicating that the actions of Syns differentially affect glutamatergic and GABAergic neurons.

Nonsense and missense mutations in *SYN1* were recently described in association with epilepsy and/or autism spectrum disorders [26]–[28]. The first such mutation was identified in a four-generation family in which some males showed an epileptic phenotype either isolated or associated with mental retardation and/or behavioral disturbances [26]. The mutation showed an X-linked recessive mode of inheritance with variable expressivity in hemizygous males and no phenotypic expression in heterozygous females. The mutation consisted in a nucleotide transition at position c.1067G>A that generates a premature translation-termination codon (PTC) in exon 9. At the protein level, this substitution (W356X) would give rise to a truncated protein missing part of the central C domain, as well as the COOH-terminal domains D and E/F. Moreover, since *SYN1* is composed of 13 exons and the PTC is more than 55 nucleotides upstream of an exon-exon junction, the possibility exists that the transcript is degraded through the nonsense-mediated mRNA decay (NMD) control mechanism, which would limit the expression of the truncated protein [29]–[35].

The aim of this work is to test the impact of the G1067A transition on Syn I expression and function. Here, we show that in HeLa cells the mutant *SYN1* mRNA is a substrate for NMD and is degraded to a large extent. When the mutant protein is expressed by transfection of the human *SYN1* cDNA, it forms Triton X-100-insoluble aggregates and is unable to reach presynaptic terminals. These data suggest that the male patients bearing the G1067A/W356× mutation lack functional Syn I due to both the strongly impaired expression and the altered function of the protein that escapes NMD.

## Materials and Methods

### Ethics statement

All experiments involving animals followed protocols in accordance with the guidelines established by the European Community Council (Directive 2010/63/EU of September 22nd, 2010) and were approved by the Institutional Animal Care and Use Committee (IACUC, permission number: 467) of the San Raffaele Scientific Institute and by the Italian Ministry of Health. All efforts were made to minimize animal suffering.

### Animals

Mice were housed under constant temperature (22 ± 1 °C) and humidity (50%) conditions with a 12 h light/dark cycle, and were provided with food and water *ad libitum*. Homozygous *SynI^−/−^* mice were kindly provided by Prof. Paul Greengard (Rockefeller University, New York, NY) [36]. *SynI^−/−^* mice were re-derived on a C57BL/6N background (Charles River, Calco, Italy).

### Plasmids

PCR-amplified wild-type (WT) human *SYN1* (splicing isoform *a*) cDNA was inserted into the pCR-Blunt vector (Invitrogen, Carlsbad, CA) by EcoRI digestion. The nonsense mutation c.1067G>A was inserted by site-directed mutagenesis (QuikChange II Mutagenesis Kit, Qiagen, Milan, Italy). The cDNAs were then inserted into the pcDNA3 expression vector (Invitrogen) through HindIII/XbaI digestion. In order to create enhanced yellow fluorescent protein (EYFP) chimeras, the *SYN1* cDNAs were subcloned into the pEYFP-C3 plasmid (Clontech, Mountain View, CA). N-terminal FLAG-tagged constructs were created by inserting the *SYN1* cDNAs into the pFLAG plasmid (Sigma-Aldrich, Milan, Italy). For NMD experiments, a minigene construct was created as follows: the *SYN1* genomic sequence from intron 7–8 to the end of exon 13 was amplified from the BAC clone RZPDB737D0410D (ImaGenes, Berlin, Germany) by high-fidelity PCR and inserted downstream exon 7 of the *SYN1* cDNA in the pcDNA3 vector (by ClaI/XhoI digestion). As NMD controls, plasmids encoding the WT and NS39 forms of *HBB* (human beta-globin) gene were used as previously described [35]. An elongated *HBB* gene (WT+300) was created by the insertion of 300 nucleotides into the SalI restriction site of WT *HBB* sequence [37] and served as a control for transfection efficiency. Lentiviral vectors were produced cloning the WT or mutated EYFP-*SYN1* fragment (see above) into in the AgeI/SalI sites of the the pRRL-PGK vector [38]. All restriction enzymes were from New England Biolabs (NEB, Ipswich, MA).

### Cell culture, transfection and transduction procedures

HeLa cells were grown in Dulbecco's Modified Eagle's Medium (DMEM; Invitrogen) supplemented with 10% fetal clone III serum (FCIII; Celbio, Pero, Italy), 1% glutamine and 1% penicillin/streptomycin (P/S; Invitrogen), at 37 °C and 5% CO_2_. Cells were transfected with plasmids and minigenes through the Ca^2+^ phosphate method with standard procedures [39]. Transient transfection of small interfering RNAs (siRNAs) was done with Oligofectamine (Invitrogen) according to manufacturer's instructions.

Astroglial cell cultures were prepared from brains of Sprague Dawley rats (Charles River) at postnatal day 2, as previously described [40].

Primary neuronal cultures were prepared from the hippocampi of embryonic day 17.5 embryos from either Sprague Dawley rats (Charles River) or WT or *Syn1^−/−^* mice of either sex as described previously [41]. The pregnant animals were killed with CO_2_, and embryos were extracted and decapitated. Skulls were opened, and brains were dissected out and placed into HBSS. Hippocampi were removed under a dissecting microscope and collected. After 15 min of incubation with 0.25% trypsin in HBSS at 37 °C, the whole hippocampi were washed with HBSS to remove trypsin and then mechanically dissociated. Neurons were plated on poly-L-lysine (0.1 mg/ml; Sigma-Aldrich)-treated 24 mm glass coverslips at a density of 120,000 cells per coverslip (low-density cultures). Cells were plated in plating medium (MEM supplemented with 10% horse serum, 3.3 mM glucose, and 2 mM glutamine) and incubated for 2–4 h at 37 °C in a 5% CO_2_ humidified atmosphere to allow adhesion to the substrate. After plating, coverslips were transferred into a cell culture dish with a glia monolayer prepared as described in Kaech and Banker (2006) [40], containing hippocampal medium [MEM supplemented with 1% N2 supplement (Invitrogen), 2 mM glutamine (Invitrogen), 1 mM sodium pyruvate (Sigma-Aldrich), and 4 mM glucose] conditioned for at least 24 h. Coverslips were turned upside down, with neurons facing the glia, separated by paraffin dots. In order to transduce neurons at 4 days *in vitro* (DIV), coverslips were placed in a clean dish containing glia-conditioned hippocampal medium and incubated for 10–15 h at 37°C in a 5% CO_2_ humidified atmosphere in the presence of viral supernatant at 1–10 multiplicity of infection. After transduction, neurons were returned to the original dishes and maintained in culture in glia-conditioned medium.

### NMD assay on HeLa cells

Target sequences of siRNAs for luciferase and *UPF1* were described previously [42]. HeLa cells were transiently transfected with siRNAs using Oligofectamine (Invitrogen), in 100 mm diameter Petri dishes following manufacturer's instructions. After 48 h cells were trypsinized and seeded in 6-well plates. Twenty-four hours after splitting, cells were transfected with plasmids encoding *SYN1* minigenes or *HBB* genes (see Plasmids section), using the Ca^2+^ phosphate method. As controls for transfection, the WT *HBB* or the WT+300 plasmids were co-transfected with *SYN1* minigenes or WT/NS39 *HBB* plasmids, respectively. Forty-eight hours after transfection, cells were lysed for total RNA extraction, and Northern blot analysis was performed.

### RNA isolation and Northern blotting

Total RNA was isolated with TRIzol reagent (Invitrogen) according to manufacturer's instructions. Northern blot analysis was performed as previously described [43] using 3 µg of total RNA per lane on a formaldehyde agarose gel. In order to generate the radioactive probe, pcDNA3-*SYN1* WT was cut with SacII and HpaI and the resulting fragment of 315 bp was cut with EcoRI. This DNA sequence was inserted into pcDNA3 EcoRI/EcoRV. A second probe was created by cutting the pcDNA3-*SYN1* WT with ClaI, blunting and cutting again with PmlI. This fragment was inserted into pcDNA3 digested with EcoRV. Both plasmids were linearized with XhoI and purified using QIAquick Nucleotide removal Kit (Qiagen). cRNAs were produced with the SP6 RNA polymerase (Promega, Milan, Italy) in the presence of α-^32^P-GTP. Radioactive signals were quantified by phosphoimaging in a FLA-3000 fluorescent image analyser (Fujifilm, Tokyo, Japan). The indicated expression levels were calculated after correction for transfection efficiency. Blots were quantified either by direct radioactivity counting or by densitometric analysis of the autoradiograms (Quantity One software, BioRad, Hercules, CA) obtained in the linear range of the emulsion response.

### Cell labelling protocols and image acquisition

Standard immunofluorescence experiments were performed as previously described [44]. Briefly, cells were rinsed once with Krebs–Ringer's solution (KRH)–EGTA (in mM: 130 NaCl, 5 KCl, 1.2 KH_2_PO_4_, 1.2 MgSO_4_, 2 MgCl_2_, 2 EGTA, 25 HEPES, and 6 glucose, pH 7.4), fixed for 15 min with 4% paraformaldehyde, 4% sucrose in 120 mM sodium phosphate buffer, pH 7.4, supplemented with 2 mM EGTA. Coverslips were rinsed three times with PBS and then incubated overnight at 4 °C in a humidified chamber with the primary antibody appropriately diluted in goat serum dilution buffer (GSDB; 15% goat serum, 450 mM NaCl, 0.3% Triton X-100, and 20 mM sodium phosphate buffer, pH 7.4). Specimens were then washed three times with PBS and incubated with the appropriate secondary antibody for 90 min at RT. After three washes with PBS for 30 min, coverslips were mounted with 70% glycerol in PBS supplemented with phenylenediamine (1 mg/ml) as an anti-bleaching agent. TRITC-conjugated phalloidin (Sigma-Aldrich) was added during incubation of the secondary antibodies when indicated. HeLa cells were stained with antibodies directed towards Syn I (G177 or G143 polyclonal antisera) [45], early endosomal antigen 1 (EEA1; BD Biosciences, Franklin Lakes, NJ), mannose-6-phosphate receptor (M6PR; Abcam, Cambridge, UK), lysosomal-associated membrane protein 1 (LAMP1; Calbiochem, Darmstadt, Germany), protein disulphide isomerase (PDI; Stressgene Biotechnologies, Victoria, BC), transferrin receptor (TfR; Zymed Laboratories, San Francisco, CA), microtubule-associated protein light chain 3 (LC3; Santa Cruz, Santa Cruz, CA), Golgi SNARE 28 (GS28; BD Biosciences Europe, Erembodegem, Belgium) and FLAG tag (Sigma-Aldrich). Neurons were stained with a monoclonal antibody directed against vesicle-associated membrane protein 2 (VAMP2; Synaptic Systems, Gottingen, Germany). Epifluorescence images were acquired with an inverted microscope (Axiovert 135; Carl Zeiss, Oberkochen, Germany) equipped with epifluorescence 63× optics. Images were recorded with a C4742-98 ORCA II cooled charge-coupled device camera.

### Polyubiquitination assay

HeLa cells were transfected with the WT or G1067A forms of the pFLAG-*SYN1* plasmid. Eight hours after transfection, cells were treated overnight with the proteasome inhibitor MG132 (Calbiochem) at a final concentration of 10 µM. Cells were lysed 24 h after transfection in lysis buffer containing either Triton X-100 (1% TX-100, 150 mM NaCl, 20 mM Tris-HCl, pH 7.4) or SDS (1% SDS, 2 mM EDTA, 10 mM HEPES, pH 7.4), supplemented with a protease inhibitor cocktail (Sigma-Aldrich). Protein concentrations were determined with the bicinchoninic acid (BCA) assay (Pierce, Rockford, IL). Immunoprecipitation of Syn I was performed overnight at 4 °C from 100 µg of protein lysates, with a polyclonal anti-FLAG antibody (Sigma-Aldrich) previously incubated with agarose beads carrying immobilized protein A (GE Healthcare, Waukesha, WI). Polyubiquitination of the samples was evaluated by Western blotting with a monoclonal antibody against ubiquitin (P4D1; Santa Cruz). The same nitrocellulose filter was stripped and incubated with the anti-FLAG antibody (Sigma-Aldrich) in order to reveal the tagged Syn I protein. SDS-PAGE was performed according to Laemmli [46]. Immunoblotting was performed using peroxidase-conjugated secondary antibodies (Bio-Rad, Hercules, CA). Detection was performed with enhanced chemiluminescence reaction (ECL; Amersham-GE Healthcare, Buckinghamshire, UK).

## Results

### The G1067A-*SYN1* mRNA is degraded by NMD and the mutant Syn I protein is expressed at very low levels in HeLa cells

**Figure 1:**
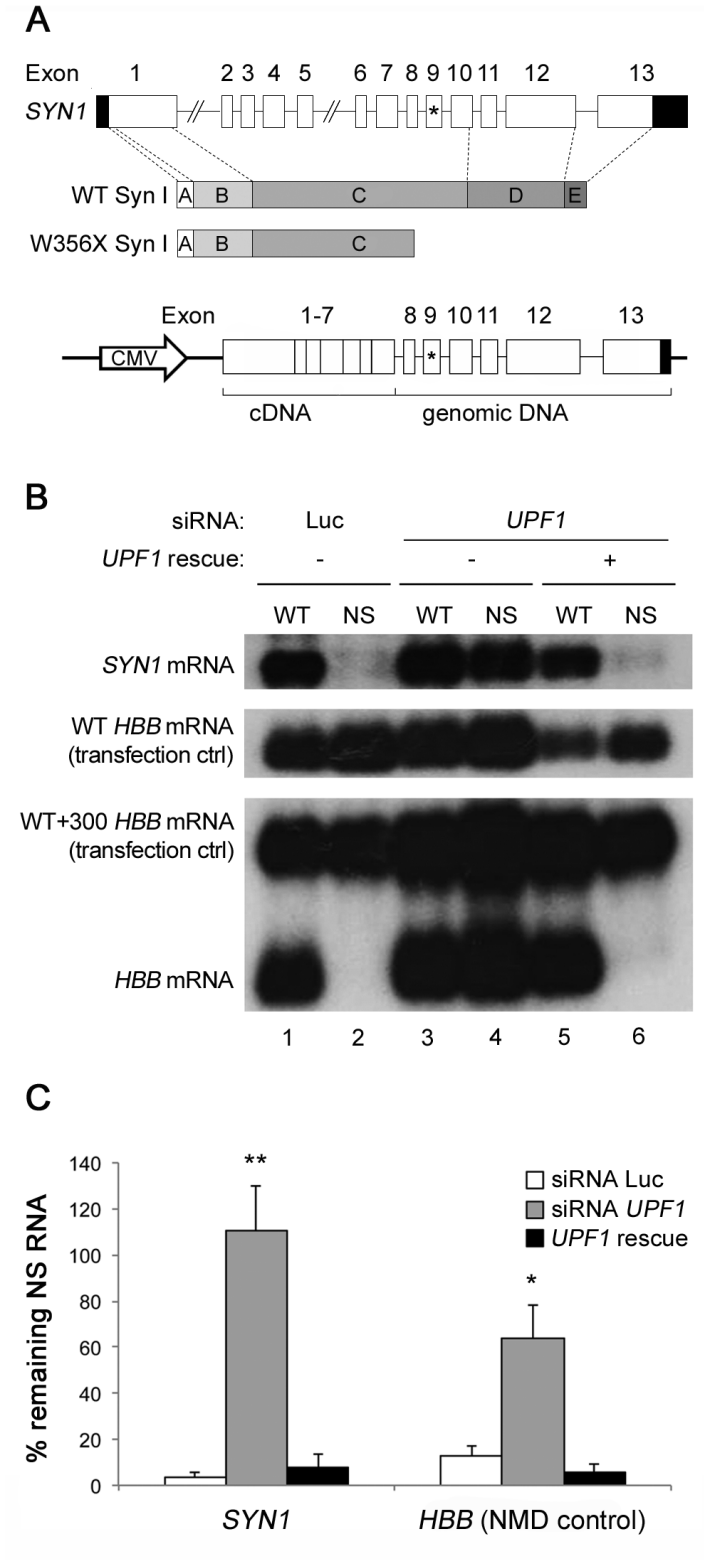
The *SYN1* transcript carrying the G1067A nonsense transition is subjected to NMD in HeLa cells. **A**. Schematic representation of the *SYN1* gene, the Syn I protein (WT and W356× variants) and the minigene construct. The position of the G1067A mutation is indicated by an asterisk in exon 9. **B**. NMD assay performed in HeLa cells. *Top and middle panels*: HeLa cells were transfected with siRNAs for luciferase (Luc; lanes 1–2) or *UPF1* (lanes 3–4). As a rescue control, cells transfected with the siRNA for *UPF1* were co-transfected with a *UPF1* cDNA construct carrying silent mutations that render it insensible to knocking-down (*UPF1* rescue; lanes 5–6). For these three conditions, cells were co-transfected with either the WT or nonsense (NS) *SYN1* minigenes and with a WT *HBB* coding plasmid (as a transfection control). When UPF1 is present, most of the mutant *SYN1* mRNA is degraded. *Bottom panel*: as a control for NMD efficiency, the same experiment was performed with the WT and NS39 variant of the *HBB* gene, with the WT+300 *HBB* construct as a transfection control. **C**. Quantification of the Northern blot analysis. Values were calculated from signal intensities according to the ratio (C_WT_ · S_NS_)/(C_NS_ · S_WT_), where S_WT_ and S_NS_ are the WT and NS samples, respectively, and C_WT_ and C_NS_ are the respective transfection controls. Data represent the mean (± SD) percentage of remaining nonsense mRNA of n = 3 independent experiments. Statistical analysis was carried out by one-way ANOVA, followed by the post-hoc Tukey's multiple comparison test (*, p<0.05; **, p<0.01).

The c.1067G>A mutation identified by Garcia and collaborators in 2004 [26] results in a nonsense change at codon 356 in the 9^th^ exon (W356X). Translation of the transcript would generate a truncated protein that lacks its COOH-terminal portion, namely part of domain C and domains D and E/F (Figure 1A) [8], [11]. This truncated protein probably misses many of the physiological properties of WT Syn I. However, a likely possibility is that the transcript carrying the PTC is subjected to NMD. In this case, protein translation would be strongly limited, thus making the *Syn1^−/−^* mouse a suitable model of the pathology.

The NMD machinery requires that at least one exon junction complex (EJC) is deposited on the target mRNA in order to be able to recognize a PTC. Therefore, WT and G1067A mutant *SYN1* minigenes containing introns upstream and downstream of exon 9, where the mutation is located, were generated. Since the *SYN1* gene consists of 50 kb and is therefore too large to be inserted in commonly employed expression vectors, an intronless cDNA comprising exons 1 to 7 was fused to the intron-containing genomic sequence including intron 7 to the end of exon 13 (Figure 1A). In order to perform the NMD assay, HeLa cells were transfected with the WT or G1067A mutant minigenes in the presence of siRNAs for either luciferase (as a control) or *UPF1*, a key factor of the NMD machinery [47]. As a positive control, a parallel experiment was performed with the WT and NS39 variants of the *HBB* gene, since efficient NMD has been described for this nonsense mutant of the *HBB* mRNA [35]. Analysis of the mRNA levels by Northern blotting revealed that the WT *SYN1* mRNA was correctly transcribed, in either the presence or the absence of UPF1 (Figure 1B, lanes 1, 3 and 5). In contrast, the G1067A mutant *SYN1* transcript was reduced to very low levels under normal conditions, i.e. when the control siRNA against luciferase was used (Figure 1B, lane 2; Figure 1C, 3.5 ± 2.1% of remaining nonsense RNA *vs* WT mRNA). This reduction was indeed mediated by NMD, since knocking-down of *UPF1* led to the accumulation of the mutant transcript (Figure 1B, lane 4; Figure 1C, 110.5 ± 19.1% of remaining nonsense *vs* WT mRNA). When the levels of UPF1 were restored by the transfection of a *UPF1* rescue plasmid [42], the mutant *SYN1* transcript was again barely detectable (Figure 1B, lane 6; Figure 1C, 8 ± 5.2% of remaining nonsense *vs* WT mRNA), demonstrating the specificity of the *UPF1* RNAi. The results of the NMD assay on the WT and mutant *SYN1* transcripts completely paralleled the observations made for the mutant *HBB* control experiment, further confirming the efficiency of NMD in our system. In particular, in the presence of siRNA against luciferase, 13 ± 4.2% of NS39 *HBB* mRNA remained with respect to the WT mRNA (Figure 1B, lanes 1 and 2; Figure 1C). Upon *UPF1* knocking-down, NS39 *HBB* transcript raised to 63.5 ± 14.8% of the WT mRNA (Figure 1B, lanes 3 and 4; Figure 1C). When *UPF1* was rescued, NS39 *HBB* mRNA was decreased to 5.5 ± 3.5% of the WT mRNA (Figure 1B, lanes 5 and 6; Figure 1C).

**Figure 2:**
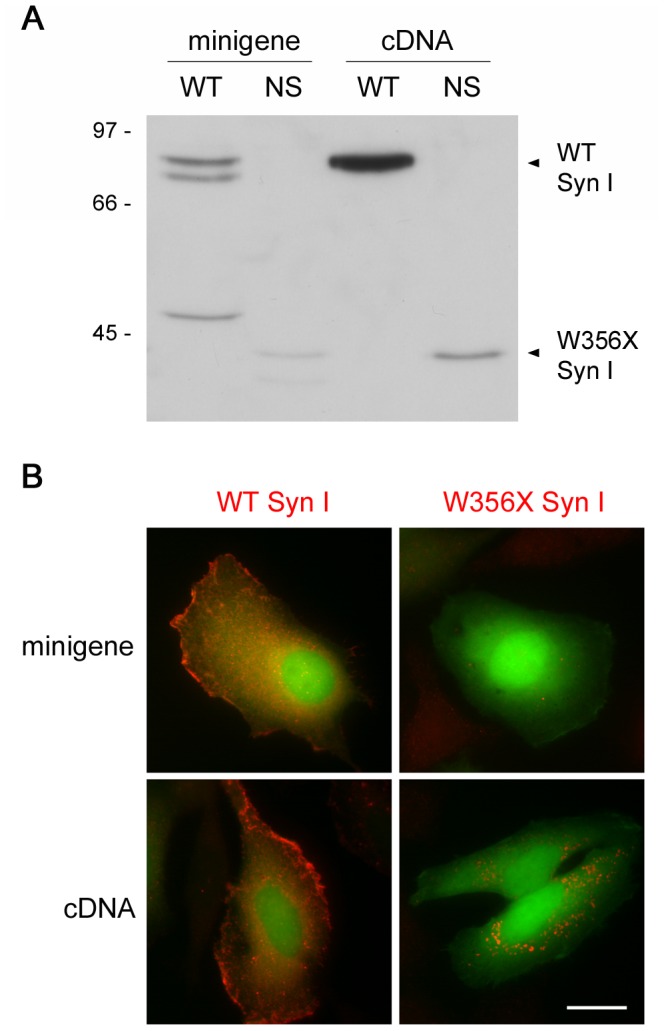
The W356× Syn I variant is poorly expressed in transfected HeLa cells. HeLa cells were transfected with either the complementary (cDNA) or genomic (minigene) DNA coding for either WT or W356× (NS) Syn I. **A**. Western blotting analysis shows that WT Syn I is efficiently expressed in both cDNA- and minigene-transfected cells (the two bands in the minigene-transfected sample corresponds to the Syn I *a* and *b* splicing isoforms). In contrast, the mutant protein is expressed with much lower efficiency in cDNA-transfected cells, and its levels are hardly detectable after transfection with the intron-containing minigene. **B**. Immunofluorescence images of HeLa cells co-transfected with pEGFP (in green) and either cDNAs or minigenes encoding either WT or mutant Syn I (anti-Syn I staining, in red). Immunostaining for mutant Syn I is appreciable in cells transfected with cDNA, but not with the minigene. Scale bar: 20 µm.

The expression of the Syn I mutant protein was also evaluated. To this aim, HeLa cells were transiently transfected with either minigenes or cDNAs coding for WT or W356× Syn I. Analysis of transfected cells by Western blotting (Figure 2A) revealed that the WT protein was efficiently expressed with either construct. As expected, in the case of the minigene, alternative splicing led to the production of the *a* and *b* Syn I isoforms. Opposite to the WT protein, the W356× mutant variant was barely detectable after transfection with the minigene, and even after cDNA transfection only a faint band could be detected. The results were confirmed by immunofluorescence of cells co-transfected with the same *SYN1* constructs and a control pEGFP vector (Figure 2B). Indeed, the signal for WT Syn I could be efficiently visualized after expression with either plasmid, while the signal for mutant Syn I was similar to the background level after minigene transfection and detectable only in few of the EGFP-positive cells when the cDNA was used. In addition, in cells positive for W356× Syn I expression, the protein formed small perinuclear aggregates (Figure 2B).

These data indicate that the biosynthesis of W356× Syn I is prevented to a large extent by NMD of the corresponding transcript. A small proportion of its mRNA may escape degradation and be translated, but the protein is probably non-functional and unstable, as it is detectable at low levels irrespective of the presence of introns that allow NMD.

### W356× Syn I forms protein aggregates that colocalize with autophagosomal markers

**Figure 3:**
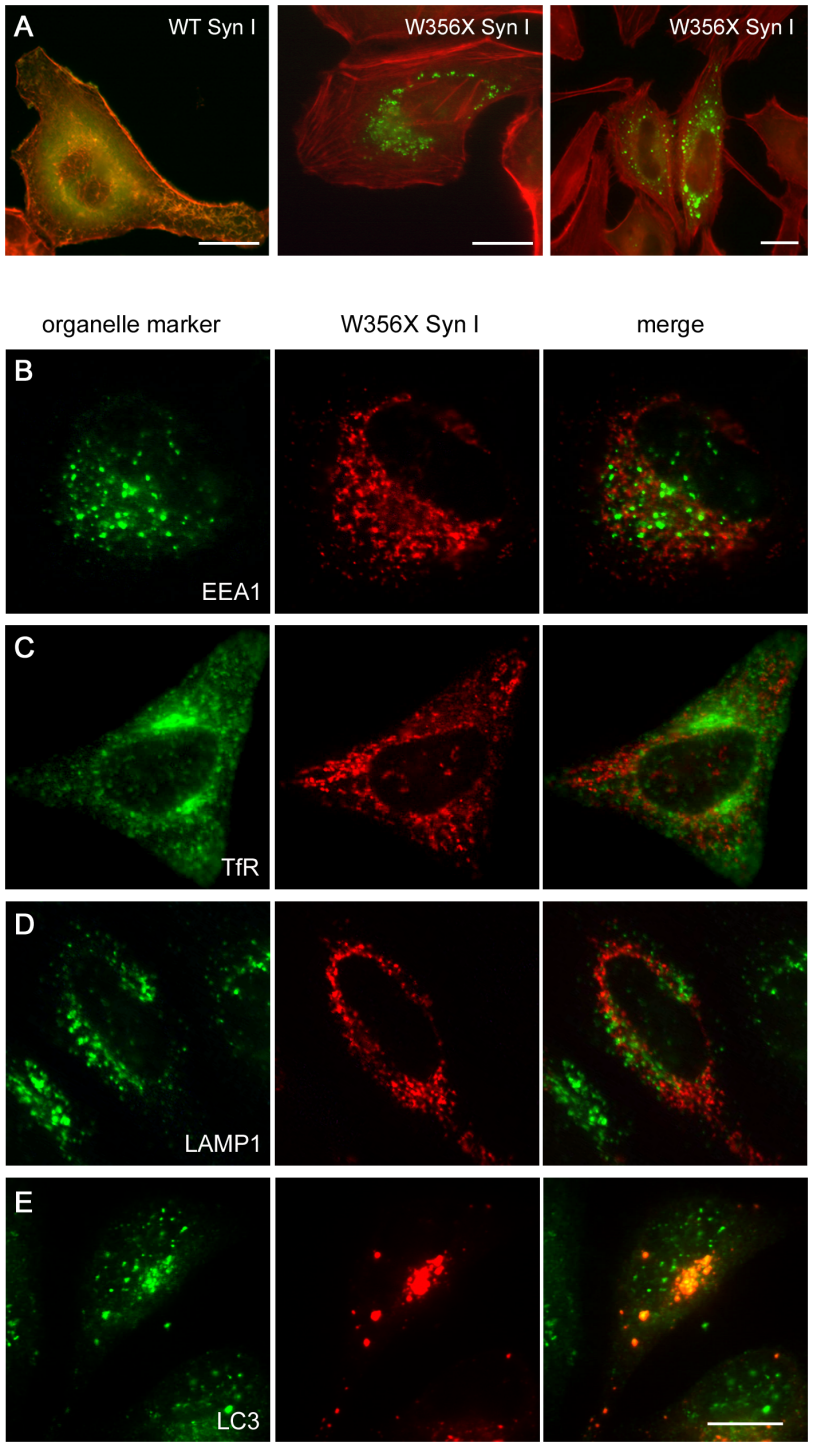
The W356× Syn I variant accumulates in aggregates that are labelled by an autophagosome marker. HeLa cells were transfected with FLAG-tagged WT or W356× Syn I coding plasmids. **A**.Representative immunofluorescence images show that the distribution of the WT protein (green) overlaps with the distribution of the F-actin filaments labelled with phalloidin (red), while the mutant form accumulates in perinuclear aggregates. WT Syn I is expressed in the majority of the cells (not shown), while W356× Syn I is only expressed by a small fraction of the cDNA-transfected cells. **B-E**.W356× Syn I aggregates (red) do not co-localize with organelle markers (green) specific for either early endosomes (EEA1; **B**), recycling endosomes (TfR; **C**) or lysosomes (LAMP1; **D**), while they co-localize with an autophagosome marker (LC3; **E**). Scale bars: 15 µm.

In order to better characterize the fate of the overexpressed mutant protein, constructs coding for FLAG-tagged Syn I were produced and transfected in HeLa cells. The distribution of the WT protein largely overlapped with the distribution of the F-actin cytoskeleton, stained with fluorescent phalloidin, as expected when the protein is expressed in non-neuronal cells [48]. On the contrary, the W356× isoform displayed cytosolic aggregates that neither associated with actin filaments nor caused gross cytoskeletal alterations (Figure 3A). To characterize the nature of these aggregates, their co-localization with specific markers of several cellular compartments was assessed (Figure 3B-E and data not shown). No co-localization was found with markers decorating the endoplasmic reticulum (PDI), the cis- and trans-Golgi network (GS28 and WGA), the early, recycling and late endosomal compartments (EEA1, TfR and M6PR) or lysosomes (LAMP1). However, the aggregates co-localized with the autophagosomal marker LC3, pointing to the possibility that the W356× Syn I mutant protein may be directed to autophagy-mediated degradation.

**Figure 4:**
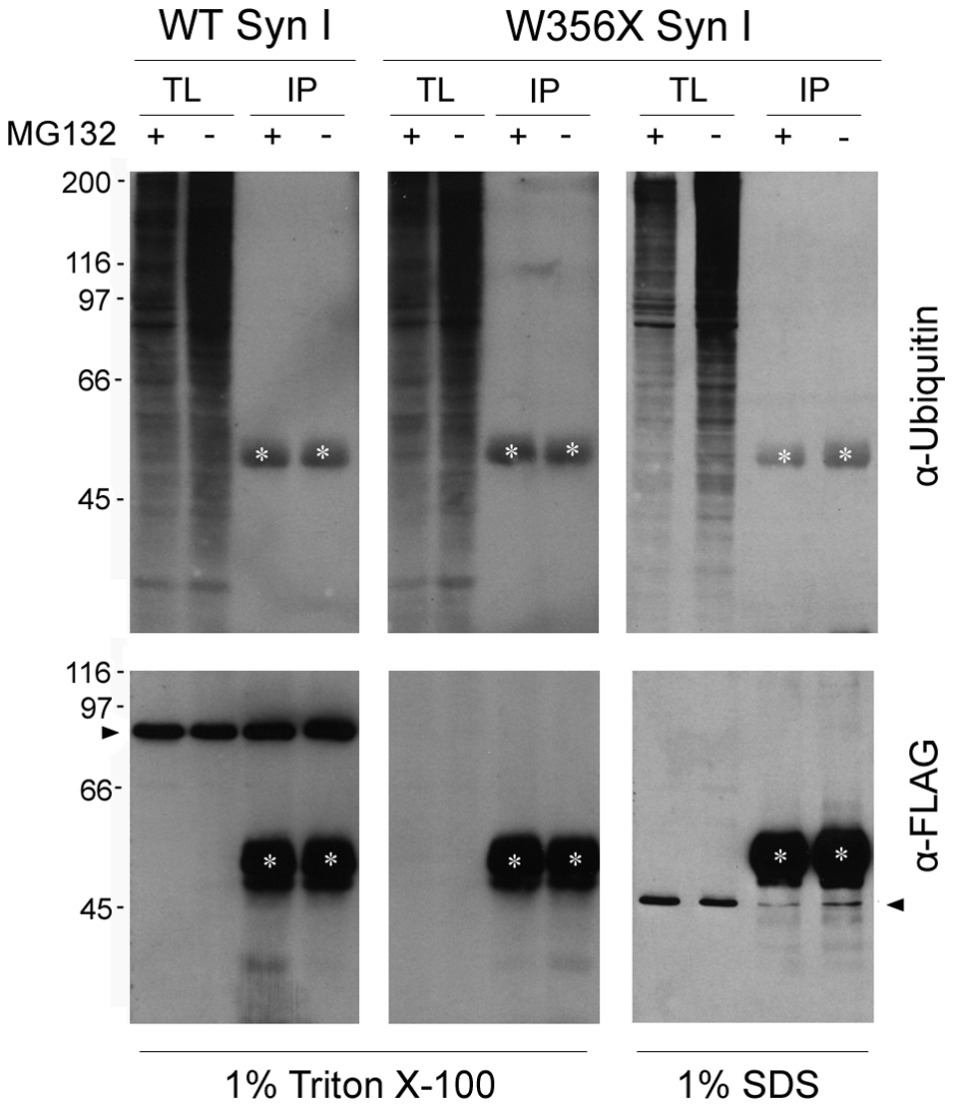
W356× Syn I aggregates are Triton X-100 insoluble and are not ubiquitinated. HeLa cells transfected with FLAG-tagged WT or W356× Syn I were lysed in 1% Triton X-100 or 1% SDS buffers after overnight treatment in the presence (+) or absence (−) of the proteasome inhibitor MG132 (1 µM). Total lysates (TL) were subjected to immunoprecipitation (IP) with an anti-FLAG antibody and the IP samples were analyzed by Western blotting with anti-Ubiquitin antibody to reveal protein ubiquitination (*upper panels*). Neither WT nor W356× Syn I appear ubiquitinated. To check for recovery of the transfected proteins after IP, the same membranes were stained with anti-FLAG antibody (*lower panels*). White asterisks indicate the IgG heavy chains. Black arrowheads indicate either the WT or W356× Syn I band.

To further analyze the possibility of increased protein degradation, we assessed the ubiquitination state of the W356× variant. The FLAG-tagged Syn I isoforms were immunoprecipitated from protein extracts of HeLa cells that had been treated or not with the proteasome inhibitor MG132. Interestingly, WT Syn I could be immunoprecipitated after lysis with Triton X-100, while aggregates of the W356× variant were insoluble in this mild detergent and the protein was detectable only upon lysis with SDS. However, we found that neither the WT nor the W356× Syn I isoforms were ubiquitinated (Figure 4). This result is in line with the evidence that Syn I is mostly degraded by calpain in the nerve terminal [49] and indicates that proteasomal degradation is not involved in lowering the expression levels of W356× Syn I.

### W356× Syn I is not targeted to nerve terminals in primary hippocampal neurons

**Figure 5:**
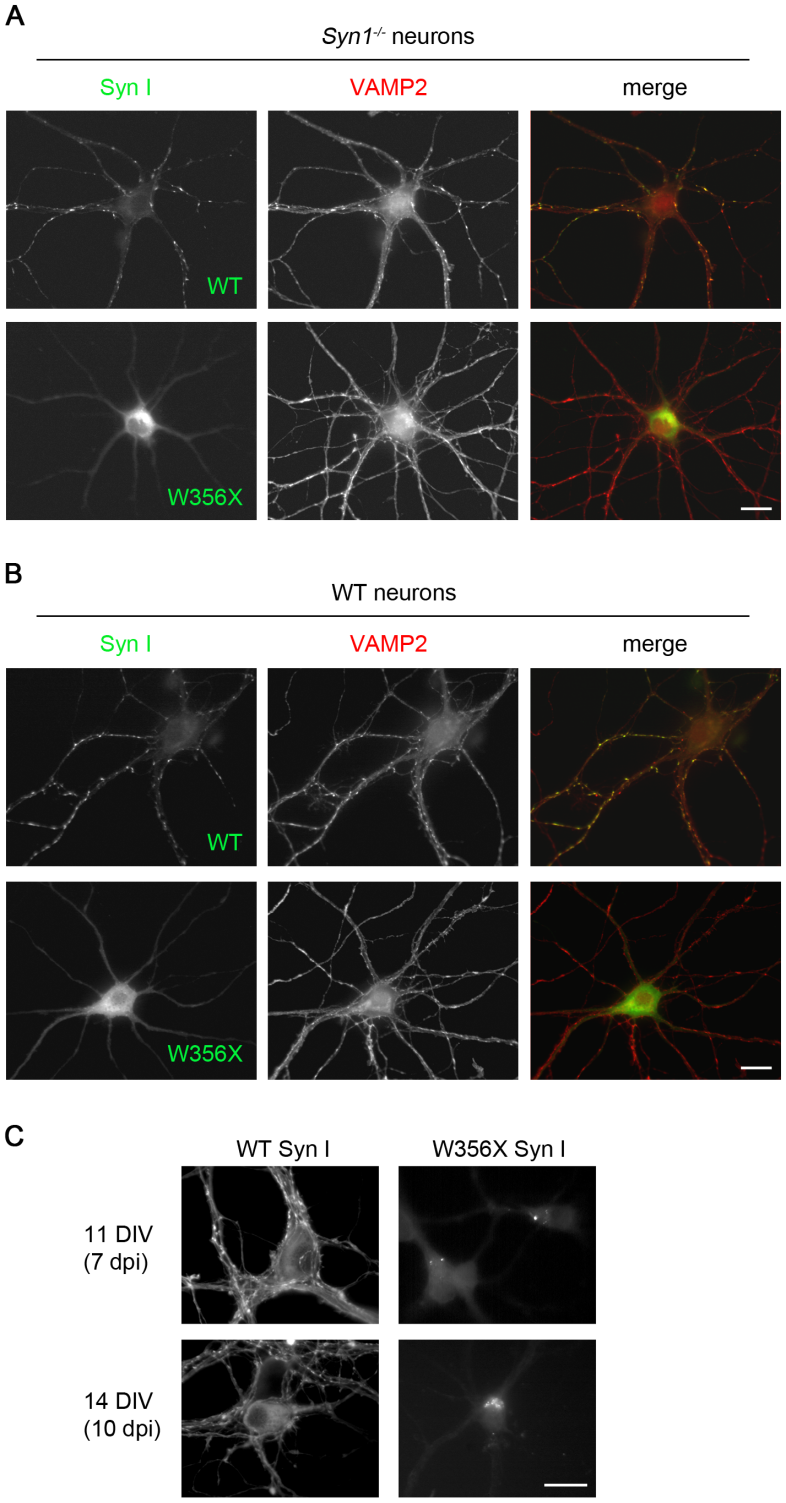
The W356× Syn I variant is not targeted to nerve terminals in either *Syn1^−/−^* or WT hippocampal neurons. **A**. *Syn1^−/−^* hippocampal neurons were transduced at 4 DIV with lentiviruses expressing EYFP-labeled either WT or W356× Syn I (green), and fixed at 8 DIV. VAMP2 (red) was used as a marker of presynaptic contacts. **B**. A similar experiment was performed in WT hippocampal neurons. In both neuronal backgrounds, WT Syn I displays a punctuate pattern overlapping with VAMP2 staining, while W356× Syn I is dispersed in the cytoplasm. The presence of endogenous Syn I in WT neurons is not sufficient to correct the mis-localisation and to redirect the mutant protein to presynaptic terminals. **C**. *Syn1^−/−^* hippocampal neurons were transduced at 4 DIV and fixed at either 11 or 14 DIV. The prolonged expression of W356× Syn I results in the formation of somatic microaggregates. Scale bars: 20 µm.

Since Syn I is a neuro-specific phosphoprotein involved in the regulation of SV exo-endocytosis at the presynaptic terminal, we investigated the ability of the mutant variant to correctly localize at synaptic boutons. To this end, we transduced 4 DIV *Syn1^−/−^* hippocampal neurons with lentiviral vectors coding for EYFP-tagged WT or W356× Syn I. Neurons were fixed and stained at 8 DIV and synaptic contacts were identified using the SV protein VAMP2 as a marker. When neurons were transduced with the lentiviral vector for the expression of EYFP-tagged WT Syn I, the fluorescent protein was identifiable in the majority of neurons and its distribution fully overlapped with that of VAMP2, pointing to a correct targeting of the exogenously expressed protein to presynaptic terminals. In contrast, in the few cells positive for expression of the EYFP-tagged W356× Syn I that we could observe, the mutant protein was uniformly dispersed throughout the neuronal cytoplasm, in the absence of any polarized distribution toward the axonal domains, at both 8 and 14 DIV (Figure 5A and data not shown).

It has been recently shown that, in the absence of Syns, SVs are dispersed throughout the axons rather than being correctly accumulated at the presynaptic terminals [50], [51]. In this context, it is noteworthy that expression of WT, but not of W356X, Syn I in *Syn1^−/−^* neurons led to partial re-clustering of VAMP2 at synaptic sites, further confirming the loss-of-function of the mutant protein.

A similar experiment was performed in WT hippocampal neurons, to verify whether the presence of endogenous Syn I was able to rescue a correct targeting of the exogenously expressed mutant Syn I, possibly through hetero-oligomerization. However, also in WT neurons W356× Syn I was unable to accumulate at presynaptic sites (Figure 5B). In both WT and *Syn1* ^−/−^ neurons, the diffused distribution of W356× Syn I was occasionally associated with the appearance of microaggregates in the neuronal cell body. Such aggregates appeared to grow in size with more prolonged overexpression (Figure 5C). In contrast, WT Syn I still retained its presynaptic localization, although it partially diffused to the pre-terminal segments of the axon when the levels of expression were exceedingly high.

## Discussion

Out of the many SV proteins that have been knocked out in mice, only the deletion of the *Syn* and *Sv2a/b* genes was found to cause an epileptic phenotype [4]–[7], [25]. Notably, *Syn1^−/−^* mice exhibit tonic-clonic generalized attacks starting from 2 months after birth. Moreover, *Syn1^−/−^* mice display defects in cognitive functions and social interactions [52], [53]. The pathogenesis of epilepsy in these mice is attributable to an imbalance between synaptic excitation and inhibition, which probably precedes the appearance of epilepsy and may trigger the process of epileptogenesis [21]–[25]. In addition, the delayed appearance of the epileptic phenotype is likely attributable to the postnatal build-up of the expression of Syn I that reaches steady-state levels at the peak of synapse formation and rearrangement [54]. While no mutations in the *SV2* genes have been found to be associated with human epilepsy to date, a first nonsense (c.1067G>A) mutation in the *SYN1* gene was described in 2004 in a four-generation family affected by a syndromic form of inherited X-linked epilepsy [26]. The affected males of the family displayed variable epileptic phenotypes (tonic-clonic seizures, nocturnal epilepsy, complex partial seizures, etc.), often associated with learning disabilities and aggressive behaviour, arising during childhood or adolescence. Additional nonsense and missense mutations in the *SYN1* gene were recently identified in families and individuals affected by epilepsy and/or autism [27], and *SYN2* was described as a susceptibility locus predisposing for disease occurrence [55], [56], thus corroborating the involvement of altered Syn function in the pathogenesis of this complex human disease.

Since a characterization of the effects of the G1067A nonsense substitution identified by Garcia and collaborators in 2004 was still lacking, we decided to focus the present work on the possible mechanisms leading to the epileptic phenotype in patients bearing this particular mutation. We identified three potential mechanisms that could lead to the loss of Syn I function in the presence of the W356× mutation: (i) degradation of the transcript; (ii) translation of a functionally inactive protein; (iii) degradation of a structurally altered protein. Indeed, we found that all the three possibilities are true and render the human pathological situation very close to the complete gene deletion modeled in the mouse. In particular, we found that: (a) the presence of a PTC in the *SYN1* mRNA leads to its marked degradation, through the surveillance mechanism of NMD; (b) the mRNA that escapes NMD translates a W356× mutant protein that in turn is probably subjected to increased degradation, as it is expressed at lower levels with respect to WT Syn I; (c) in mature hippocampal neurons grown in culture, mutant Syn I is not targeted to presynaptic terminals and accumulates in aggregates at the cell soma.

Concerning the degradation of the mutant transcript through NMD, this process is a well-characterized physiological quality-control mechanism that selectively degrades mRNAs harboring PTCs, preventing the production of truncated proteins with potential dominant-negative or toxic activities. The deposition of the multiprotein EJC during splicing and the first round of translation (termed “pioneer round”) are necessary steps for the recognition of a PTC by the NMD machinery and the triggering of mRNA degradation. In addition, the PTC is able to drive strong NMD if it is located at least 50–55 nucleotides upstream of an exon-exon junction, as in this way the EJC can act as a binding platform for the NMD factors without masking the nonsense codon [29]–[34]. The G1067A mutation, located ≈90 nucleotides upstream of the junction between exon 9 and exon 10 of *SYN1*, has the potential to make the *SYN1* mRNA a substrate for NMD. However, the occurrence of NMD cannot be assumed *a priori* based on the presence of a PTC in an appropriate location, and has to be investigated experimentally [33]. We found that in NMD-competent HeLa cells, the G1067A mutant *SYN1* transcript (produced through transfection of a minigene construct in order to allow splicing) was degraded to a large extent, similarly to what observed for the NS39 variant of the *HBB* gene, for which efficient NMD has been described [35]. Unfortunately, we were not able to perform the same experiment in a neuronal system, since the minigene plasmids were too large to be efficiently delivered into hippocampal neurons. In addition, only scarce data are available concerning the efficiency of NMD in neurons.

Secondarily, we showed that the W356× Syn I protein is prone to form aggregates in the cell body of the expressing cells. The central highly conserved C domain, in which the mutation resides, contains both hydrophobic and highly charged amino acid sequences. The domain is characterized by a compact structure, organized in three subdomains folded as α-helices and β-sheets and a disordered COOH-terminal region that together form an ATP binding site [57]. The binding of ATP is Ca^2+^-dependent and is important for the formation and stabilization of Syn I oligomers [58]. Thus, the W356× mutation has a likely effect on the secondary structure of the protein that, together with the complete deletion of the ATP binding site, may impact on Syn I oligomerization dynamics. In particular, the portion of the C domain downstream of the truncation contains 25% of highly charged (D, E, K and R) residues, and its deletion may have a destabilizing effect on the remaining C domain, promoting the formation of insoluble aggregates. We showed that these aggregates co-localize with the autophagosomal marker LC3, pointing to the fact that the protein might be directed to degradation although not mediated by the proteasome, since it was not possible to demonstrate its ubiquitination. It is also possible that the microaggregates exert a toxic function. Indeed, the precocious expression of W356× in developing neurons impairs axonal elongation and branching (M. Giannandrea, F.C. Guarnieri, E. Monzani and F. Valtorta, unpublished observations).

In order to exert their functions in the presynaptic compartment, Syns need to be anterogradely transported from the cell body to the axon terminals. Syn I is targeted to nerve terminals by fast axonal transport, through the binding to pre-assembled SVs or mobile packets containing several presynaptic components, as well as by slow axonal transport possibly in association with cytoskeletal components [59]. Both transport modalities rely on the interactions of Syn I with actin filaments and SVs or their precursors. The contribution of each domain of Syn I to presynaptic targeting has been recently defined [60]. Domains C and E are the major positive determinants for the correct localisation of Syn I at nerve terminals, consistent with the major role played by these domains in the interaction with SVs and actin and in oligomerization of the protein [17], [61]–[68]. Indeed, dimerization of Syn I was shown to be another requirement for correct synaptic targeting [60]. The W356× mutant Syn I lacks part of domain C, as well as the entire domains D and E. Their loss may be responsible for the destabilization of the binding with Syn I physiological partners, and the consequent loss of a correct presynaptic targeting. The selective targeting to presynaptic terminals was not rescued by the presence of endogenous WT Syn I, probably because of the loss of the domains involved in homodimerization of the protein. Interestingly, the Q555× Syn I mutant recently described by Fassio and collaborators [27], in which domain C and part of domain D are preserved, correctly targeted to presynaptic terminals [27], [28].

In conclusion, we showed that the G1067A nonsense mutation triggers NMD-driven degradation of a large percentage of the mutant *SYN1* mRNA. Therefore, the human condition can be explained by a complete loss of Syn I function and can thus be reliably modeled through the use of the *Syn1^−/−^* mouse. The occurrence of NMD may also exert a protective activity in heterozygous carriers of the mutation and explain the absence of a disease phenotype in these subjects, similarly to what has been observed in other diseases (i.e. β-thalassaemia) [33], [34]. Indeed, in heterozygotes the normal allele might lead to synthesis of WT Syn I in sufficient levels to ensure proper function, whereas NMD might prevent the accumulation of mutant Syn I at levels which would lead to the formation of potentially harmful aggregates.
